# Repetitive Religious Chanting Invokes Positive Emotional Schema to Counterbalance Fear: A Multi-Modal Functional and Structural MRI Study

**DOI:** 10.3389/fnbeh.2020.548856

**Published:** 2020-11-24

**Authors:** Junling Gao, Stavros Skouras, Hang Kin Leung, Bonnie Wai Yan Wu, Huijun Wu, Chunqi Chang, Hin Hung Sik

**Affiliations:** ^1^Buddhism and Science Research Lab, Centre of Buddhist Studies, The University of Hong Kong, Hong Kong, Hong Kong; ^2^Department of Biological and Medical Psychology, Faculty of Psychology, University of Bergen, Bergen, Norway; ^3^School of Biomedical Engineering, Health Science Center, Shenzhen University, Shenzhen, China

**Keywords:** emotion regulation, religious chanting, functional magnetic resonance imaging (fMRI), religious schema, brain asymmetry, amygdala

## Abstract

**Introduction:**

During hard times, religious chanting/praying is widely practiced to cope with negative or stressful emotions. While the underlying neural mechanism has not been investigated to a sufficient extent. A previous event-related potential study showed that religious chanting could significantly diminish the late-positive potential induced by negative stimuli. However, the regulatory role of subcortical brain regions, especially the amygdala, in this process remains unclear. This multi-modal MRI study aimed to further clarify the neural mechanism underlying the effectiveness of religious chanting for emotion regulation.

**Methodology:**

Twenty-one participants were recruited for a multi-modal MRI study. Their age range was 40–52 years, 11 were female and all participants had at least 1 year of experience in religious chanting. The participants were asked to view neutral/fearful pictures while practicing religious chanting (i.e., chanting the name of Buddha Amitābha), non-religious chanting (i.e., chanting the name of Santa Claus), or no chanting. A 3.0 T Philips MRI scanner was used to collect the data and SPM12 was used to analyze the imaging data. Voxel-based morphometry (VBM) was used to explore the potential hemispheric asymmetries in practitioners.

**Results:**

Compared to non-religious chanting and no chanting, higher brain activity was observed in several brain regions when participants performed religious chanting while viewing fearful images. These brain regions included the fusiform gyrus, left parietal lobule, and prefrontal cortex, as well as subcortical regions such as the amygdala, thalamus, and midbrain. Importantly, significantly more activity was observed in the left than in the right amygdala during religious chanting. VBM showed hemispheric asymmetries, mainly in the thalamus, putamen, hippocampus, amygdala, and cerebellum; areas related to skill learning and biased memory formation.

**Conclusion:**

This preliminary study showed that repetitive religious chanting may induce strong brain activity, especially in response to stimuli with negative valence. Practicing religious chanting may structurally lateralize a network of brain areas involved in biased memory formation. These functional and structural results suggest that religious chanting helps to form a positive schema to counterbalance negative emotions. Future randomized control studies are necessary to confirm the neural mechanism related to religious chanting in coping with stress and negative emotions.

## Introduction

Religious chanting and praying has existed throughout the history of the civilization of humankind, in both Eastern and Western societies and in ancient and modern times. Despite its universal popularity, very few studies have ever attempted to explore the underlying neural mechanism (Rao et al., 2018; Gao et al., 2019). Religious activities are generally not regarded as rational or logical, from a scientific point of view. At the same time, science challenges religious beliefs through the prevalence of reductionism and biological determinism (Purvis, 2013). One practical approach is to investigate the psychological benefits of religious activities to understand how religious activities might help individuals to confront hardship (Taylor, 2001). We propose that the foremost potential application of religious chanting could be in alleviating the symptoms of affective distress because various religious activities are associated with existential and emotional thinking, rather than with rationality and formal reasoning (Corrigan, 2002; Graves, 2008). Thus the neural correlates of religious chanting may be associated with affective processes and corresponding structural brain changes (Corrigan, 2008; Davies, 2013). However, the majority of previous related studies explored behavioral aspects, while few investigated the psychological benefits of religious practice from a neuroscientific perspective (Harris et al., 2009; Kim-Prieto and Diener, 2009; Alcorta, 2020).

In a previous functional magnetic resonance imaging (fMRI) study, Wiech et al. (2008) found that religious contemplation can help individuals to reinterpret the emotional significance of pain, making it easier for them to detach themselves from the experience of pain. Pain modulation is proportional to the degree of liking stimuli comprised of religious images (Wiech et al., 2008). Religious thinking appears to be related to brain regions responsible for emotion, self-representation, and cognitive conflict (Harris et al., 2009). A previous electroencephalogram (EEG) study comparing different meditative practices found lower coherence during meditation and globally reduced functional interdependence between brain regions, which may help practicing individuals to experience detachment and let go (Lehmann et al., 2012). Religious beliefs can also influence self-reflection (Wu et al., 2010).

Despite the extreme popularity of religious chanting and prayer, no previous study has directly investigated the mechanism of repetitive religious chanting (RC) on negative emotion modulation. The present study can help to explain the positive effects of religious practices from a neuroscientific perspective. The human brain is susceptible to negative events and a “negativity bias” has developed during our evolution, to protect us. This negativity bias aids survival, but in extreme cases, it may contribute to negative rumination and even depression (Rude et al., 2002). Different methods have been developed to ameliorate negativity, including music, exercise, meditation, chanting or praying, and other non-pharmaceutical methods (Orini et al., 2010; Tang et al., 2016).

According to religious practitioners, in contrast to our habitualized pattern of negativity bias and biologically hardwired modes of fight or flight, religious practice may cultivate a profound sense of peace and reassurance, and recalling religious and spiritual experiences can evoke special feelings of awe. Religious awe has long been pondered by religious leaders and philosophers, and it is assumed to have the ability to change our feeling and perception of the world (Keltner and Haidt, 2003; Gottlieb et al., 2018). In East Asia, repetitive RC is one of the most popular forms of religious practice, and it is assumed to foster a stable mental state for monks and believers alike. However, this practical method of emotion regulation has not received sufficient attention from a neuroscientific perspective. The potential effect of religious practice on emotion regulation abilities can be hypothesized given recent studies which demonstrated that the amygdala, a critical brain region for affective processes, does not only respond to fearful stimuli but also to positive stimuli and neural processing related to morality (Mercadillo et al., 2007; Cunningham and Kirkland, 2014).

Overall, studies suggest that the amygdala is most responsive to negative stimuli such as fear and disgust and that it plays a critical role in emotion processing (Costafreda et al., 2008). More recently, human and non-human primate studies found that the amygdala was engaged in Pavlovian learning (Bernardi and Salzman, 2019) and in endowing cognitive schemas (e.g., words, concepts, rules, and conclusions) with emotional valence (Murray et al., 2009). The left and right amygdalae may play different roles in emotional information processing. For example, electrical stimulation of the left amygdala induces either unpleasant feelings (fear, anxiety, or sadness) or pleasant feelings (happiness), while electrical stimulation of the right amygdala induces negative emotions only, such as sadness and fear (Cinar et al., 2016). Although the left and right amygdalae display anatomical connections and functional interactions, differences in their functions have been suggested. The right amygdala mediates autonomic stimulus detection, and the left amygdala plays a more evaluative and discriminative role in the processing of emotional stimuli (Glascher and Adolphs, 2003; Costafreda et al., 2008). In addition to the amygdala, other subcortical structures also play essential roles in affective processing, and the hippocampal complex in particular is involved in processing memories of emotional events (Yang and Wang, 2017).

Subcortical regions interact with neocortical regions during emotion regulation, both directly and indirectly, underscoring the complexity of processes related to the modulation of emotion and memory (Grossberg, 2009). The frontal lobe plays a vital role in several emotion-regulation strategies, including cognitive change, distraction, and suppression (Dorfel et al., 2014). The interaction between the amygdala and the prefrontal lobe may underlie the strategic cognitive change in emotion regulation (Banks et al., 2007) and its malfunction may lead to affective disorders (Zhang et al., 2018; Duggirala et al., 2019). Cognitive change as an emotion regulation strategy is supported by the embodied psycholinguistic theory stating that cognition can be constructed (Ellis, 2019; Sevinc and Lazar, 2019) and interestingly, the “Mind-Only” school of Yogācāra Buddhism, also proclaims that human awareness or consciousness can be actively altered by mental training (Tagawa and Muller, 2009). In fact, a previous study suggested that religious practices may promote cognitive change in relation to fearful events (McIntosh, 1995).

RC may engage a distinct neural mechanism compared to other emotion regulation strategies, such as mindfulness, which are capable of regulating negative emotions effectively (Lutz et al., 2014). In contrast to mindfulness, RC is sometimes accompanied by religious awe, an overwhelming feeling with a supernatural element (Van Cappellen and Saroglou, 2012), that indicates that RC may have a unique feature related to emotion regulation. In exploring traditional and effective methods of emotion regulation, previous event-related potential (ERP) study showed that RC of Amitābha Buddha tends to reduce the late positive potential (LPP) induced by fear- and stress-provoking pictures (Gao et al., 2017). However, affective processing relies on subcortical brain regions, such as the amygdala, whereas ERPs can only measure brain activity on the surface of the scalp. The present MRI study was conducted to investigate the role of subcortical regions, especially the amygdala, in regulating emotion through RC.

## Materials and Methods

The study was approved by the Human Research Ethics Committee of the University of Hong Kong. Twenty-one participants were recruited and signed written consent forms before participating in the experiment. Each participant was given $200 HKD as a compensation for their time and transportation costs. We used the Beck Depression Inventory (BDI) and interviews to exclude participants with depression or any other neuropsychiatric pathology. The participants were all Buddhists but belonged to different sects. They all had at least 1 year of experience in chanting the name of the Amitābha Buddha.

**FIGURE 1:**
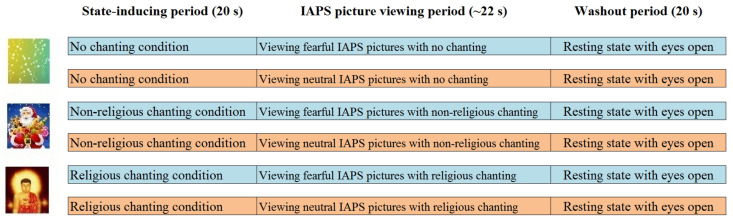
Experimental paradigm. The first 20 s period was a state-inducing chanting period (one of the three chanting conditions: RC, NRC, or NoC). In the second 20 s period, the participants continued to chant while viewing a block of IAPS pictures (either fearful or neutral in each trial). The third 20 s period was a resting state, to allow the neural activations to return to baseline.

The fMRI experiment on emotion regulation followed a previous ERP experiment (Gao et al., 2017). It had a 2 × 3 factorial design with two factors (emotion and chanting), featuring two levels for emotion (neutral and fear) and three levels for chanting (religious chanting, non-religious chanting and no chanting). A state-inducing period preceded each main experimental trial and the main trials featured the viewing of picture stimuli from the international affective picture system (IAPS; Coan and Allen, 2007), across the three levels of chanting. However, the state-inducing periods also served as different levels, to compare the brain activity between the three chanting conditions in the absence of emotion stimuli from IAPS (see conditions 1–3 below). Thus, as shown in Figure 1, the experimental paradigm featured nine conditions overall: (1) religiously chanting “Amitābha” while looking at a picture of Amitābha Buddha (RC), (2) non-religious chanting of “Santa Claus” while looking at a picture of Santa Claus (NRC), (3) no chanting while looking at an abstract color-balanced picture (NoC), (4) religiously chanting “Amitābha” while looking at a block of 10 fearful IAPS pictures (RC-Fear), (5) non-religious chanting of “Santa Claus” while looking at a block of 10 fearful IAPS pictures (NRC-Fear), (6) no chanting while looking at a block of 10 fearful IAPS pictures (NoC-Fear), (7) religiously chanting “Amitābha” while looking at a block of 10 neutral IAPS pictures (RC-Neut), (8) non-religious chanting of “Santa Claus” while looking at a block of 10 neutral IAPS pictures (NRC-Neut), and (9) no chanting while looking at a block of 10 neutral IAPS pictures (NoC-Neut). Chanting was performed mentally, internally, and silently, without moving the mouth, following the common practice for practitioners of religious chanting. The nine conditions were presented in triplets, consisting of three periods: a 20 s induction period featuring one of conditions 1–3, followed by a 20 s period with one of conditions 4–9 but featuring the same chanting condition as the preceding induction period, and finally a 20 s resting state period. Each block of IAPS picture-viewing featured 10 IAPS pictures lasting approximately 2 s each, including an interstimulus interval between the presentations of the IAPS pictures with a jitter of up to 200 ms.

Participants were asked to keep the same pace for both RC and non-RC and the trials were split into three sessions. After the scanning, the participants were asked to provide their subjective assessment regarding the three pictures used for the state-inducing periods (see Figure 1); that is, the participants’ subjective assessment regarding the picture of a religious figure (Amitābha Buddha), a non-religious figure (Santa Claus), and the color-balanced control picture (see Figure 1 and Supplementary Tables 1, 2). The results of these ratings showed that participants had strong faith in their religious figure, in contrast to Santa Claus (see Supplementary Table 2). This ensured the suitability of the chanting for inducing and sustaining the corresponding prayer-like state during the subsequent emotion regulation period. For further methodological details, please refer to a previous study using the same paradigm (Gao et al., 2017).

The data were collected using a 3T Philips MRI scanner. An anatomical T1 image was acquired with the following parameters: acquisition matrix 256^∗^256; FoV 256^∗^150^∗^240; TR 15 ms; TE 3.26 ms; voxel resolution 0.94^∗^1.0^∗^1.5 mm^3^. Gradient echo-planar imaging (EPI) was performed in the fMRI session. An eight-element SENSE head coil was used. fMRI parameter settings were as follows: acquisition matrix 64^∗^64; FoV 230^∗^140^∗^230 mm; number of slices 32; slice thickness 3 mm; slice gap 1.5 mm; TR 2,000 ms; TE 30 ms; flip angle 90°. The entire fMRI session featured 540 dynamic volumes. Data collection for the fMRI experiment lasted approximately 20 min (three sessions, each 6 min, plus inter-session rest) and for the MRI T1 scan approximately 8 min.

There were 10 IAPS pictures in each block-design trial and 3 blocks per condition. Data analysis followed the standard steps of statistical parametric mapping (SPM12, Wellcome Department of Cognitive Neurology, London). The individual fMRI data were preprocessed using standard methods of realignment, co-registration to the anatomical image, normalization, and smoothing. The preprocessed data were entered into a first-level statistical model resulting in nine contrast images, that were all computed in comparison to resting-state as baseline, corresponding to the following effects: (1) RC while viewing fearful IAPS stimuli (RC-Fear), (2) RC while viewing neutral IAPS stimuli (RC-Neut), (3) NRC while viewing fearful IAPS stimuli (NRC-Fear), (4) NRC while viewing neutral IAPS stimuli (NRC-Neut), (5) NoC while viewing fearful IAPS stimuli (NoC-Fear), (6) NoC while viewing neutral IAPS stimuli (NoC-Neut), (7) RC, (8) NRC, and (9) NoC. In the second level, the contrast images corresponding to contrasts 1–6 were entered into one random-effects general linear model (GLM) and the images corresponding to contrasts 7–9 were entered in another random-effects GLM, resulting in the following second-level contrasts: (1) fear > neutral during RC, (2) fear > neutral during NRC, (3) fear > neutral during NoC, (4) RC, 5) NRC, and (6) NoC (see Table 1 and Supplementary Figures 1, 2).

**TABLE 1:** Brain regions that presented higher brain activity during fearful IAPS picture viewing (compared to neutral IAPS picture viewing), split according to the three levels of the religious chanting, condition (*p* < 0.001, uncorrected).

Anatomical label (AAL)	Peak *T*-value	Voxels	Co-ordinates		
**Religious chanting (RC-Fear > RC-Neut)**					
Thalamus_L	4.71	1,089	−18	−28	6
Thalamus_R	4.09	234	24	−28	2
Amygdala_L	4.05	244	−22	2	−22
Amygdala_R	3.48	28	26	−2	−14
Parahippocampal_L	3.6	55	−26	−26	−18
Occipital_inf_L	5.48	1,414	−46	−76	−4
Temporal_inf_R	4.93	965	52	−66	−10
Frontal_sup_medial_L	3.84	48	2	60	28
Parietal_sup_L	4.67	170	−34	−52	56
Precuneus_R	3.97	165	6	−48	14
Cingulum_post_L	3.64	43	−8	−36	28
Sup_semilunar_R (Cerebelum_6_R)	3.62	39	12	−66	−28
Cerebelum_tonsil_R (Cerebelum_9_R)	4.42	304	10	−52	−38
Pons_L	6.01	133	−6	−33	−30
**Non-religious chanting (NRC-Fear > NRC-Neut)**					
Fusiform_R	4.52	60	48	−34	−18
Occipital_mid_L	4.16	116	−46	−62	2
**No chanting (NoC-Fear > NoC-Neut)**					
Temporal_inf_L	3.72	27	−50	−68	−8

*L, left; R, right; Inf, inferior; Mid, middle; Post, posterior; Sup, superior.*

### ROI Analysis

Region of interest (ROI) analysis was applied to the amygdala because of its involvement in affective processes. Moreover, a correlation analysis was performed using levels of amygdala activation in the two emotion conditions, during religious chanting, to investigate the effect of RC on amygdala responsiveness to affective stimuli.

The amygdala activity was calculated using the Marsbar SPM toolbox^1^. The ROIs of the left and right amygdalae were defined by default in Marsbar, according to the Montreal Neurological Institute (MNI) Anatomical Automatic Labeling (AAL) template.

Following the standard Marsbar data analysis process, the individual fMRI data and the first-level results, obtained as described above, were used to compute ROI percentage signal change for each side of the amygdalae. The fMRI statistics for each type of picture and chanting condition were calculated for each side of the amygdalae and for every participant. The results were analyzed with a one-way within subjects ANOVA (repeated measurements), using SPSS 20^2^.

### Brain Asymmetry Analysis

The 12-step asymmetries voxel-based morphometry (VBM) analysis method (Kurth et al., 2015) was performed on the T1 MRI, by flipping the anatomical images and calculating the asymmetry index (AI) based on the formula: $\text{AI}=\,\frac{\left(\text{original}-\text{flipped}\right)}{0.5\,\left(\text{original}+\text{flipped}\right)}$. This produced AI maps with the right hemisphere masked by the flipped version of the left hemisphere for each individual subject, to enable their morphometric comparison. The corresponding data processing steps were performed using the SPM8^3^, vbm8^4^, and Mricron^5^ software packages.

## Results

**FIGURE 2:**
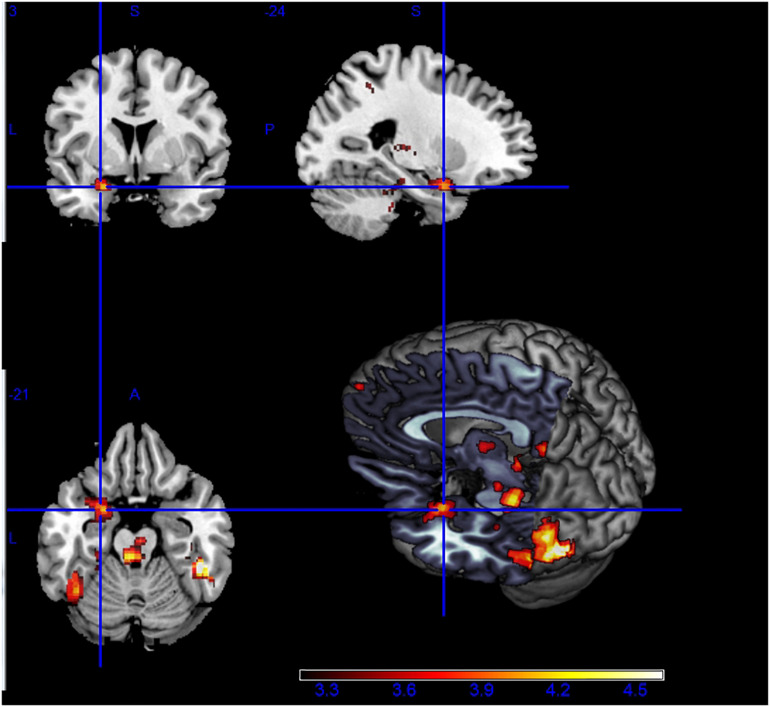
Higher brain activity during the viewing of fearful vs. neutral pictures, while being engaged in religious chanting (i.e., RC-Fear > RC-Neut). Asymmetric activation between **right** and **left** amygdala is visible (*p* < 0.001, uncorrected).

The fMRI results showed higher brain activity in certain regions during RC-Fear, compared with the RC-Neut. The related regions included regions in the fusiform gyrus, bilateral occipital lobes, prefrontal lobes, as well as the thalamus, amygdala, para-hippocampus, and cerebellum. Asymmetric amygdala activity was observed during RC, with significantly higher activity in the left than the right amygdale (see Figure 2). In general, during non-religious chanting and no chanting, results were similarly localized, although considerably weaker (see Supplementary Figure 1).

Brain regions related to the processing of fear during the three chanting conditions are detailed in Table 1.

**FIGURE 3:**
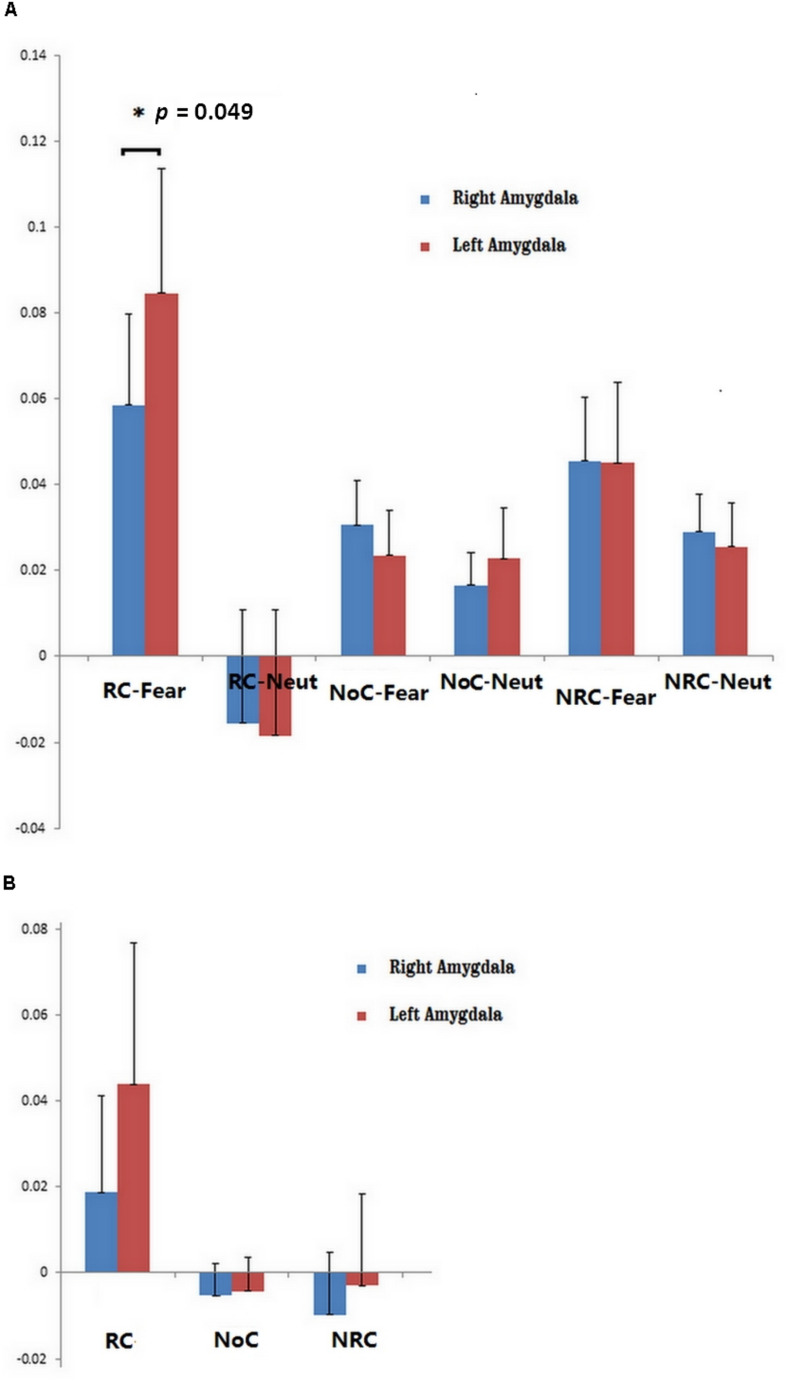
**(A)** ROI analysis of left and right amygdala activity during six conditions: RC-Fear, religious chanting while viewing fearful pictures; RC-Neut, religious chanting while viewing neutral pictures; NoC-Fear, no chanting while viewing fearful pictures; NoC-Neut, no chanting while viewing neutral pictures; NRC-Fear, non-religious chanting while viewing fearful pictures; and NRC-Neut, non-religious chanting while viewing neutral pictures (*p* < 0.05, uncorrected). **(B)** ROI analysis of left and right amygdala activity during three conditions: RC, religious chanting without viewing IAPS pictures; NoC, no chanting without viewing IAPS pictures; and NRC, non-religious chanting without viewing IAPS pictures. The error bars represent the standard error of the mean.

Subcortical activity in regions including the thalamus, amygdala, cerebellum, and brainstem was stronger during RC than NRC. More importantly, activity in the left amygdala was higher than activity in the right amygdala in RC-Fear (*p* = 0.049, uncorrected) (see Figure 3). Similar asymmetry was also observed during the RC condition (without IAPS picture viewing).

**FIGURE 4:**
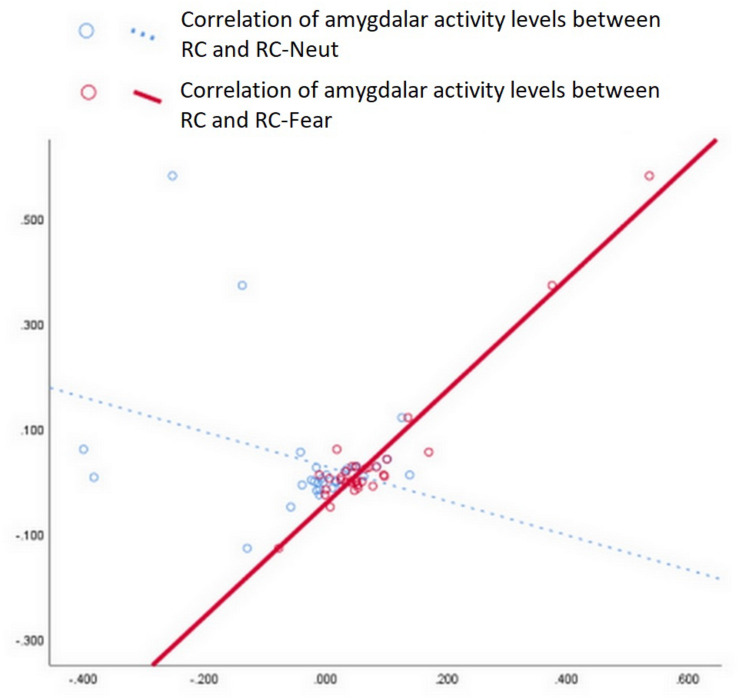
Correlation analysis of amygdala values between RC and RC-Fear, as well as between RC and RC-Neut. A strong positive correlation was only significant between RC and RC-Fear (*p* < 0.001, Bonferroni uncorrected).

To investigate the effect of RC on probing the responsiveness of the amygdala, we performed a Pearson correlation analysis. This analysis explored the linear correlation in amygdala activation levels between the inducing periods and the corresponding stimulation periods (e.g., between RC and RC-Fear, or between RC and RC-Neut) (see Figure 4).

We noticed a strong positive correlation between amygdala activity during RC and RC-Fear, with *r* = 0.960 (*p* < 0.001, Bonferroni corrected). The correlation between amygdala activity during RC and RC-Neut was *r* = −0.321 (*p* = 0.073, uncorrected).

**FIGURE 5:**
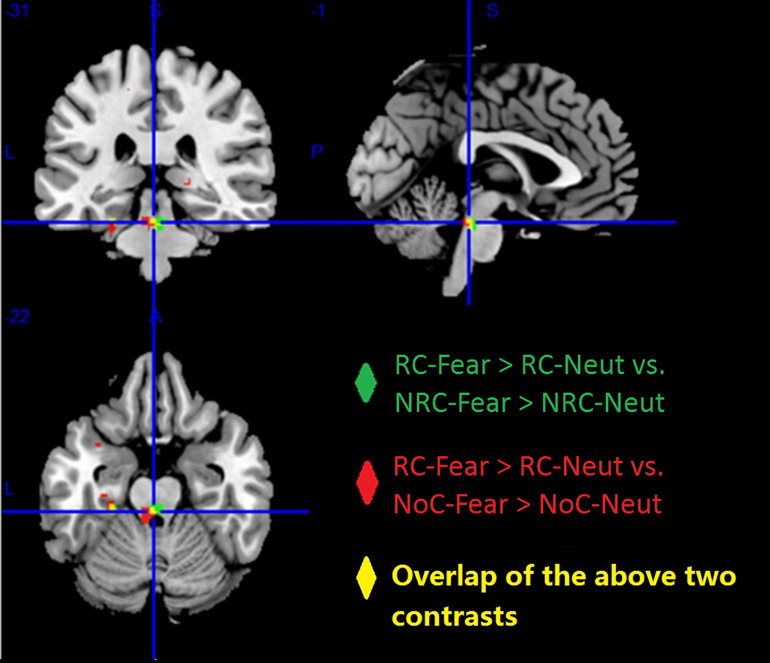
Higher brain activity was observed for the contrast “RC-Fear > RC-Neut,” compared to “NRC-Fear > NRC-Neut” (green), and “NoC-Fear vs. NoC-Neut” (red). The overlap between these two comparisons (yellow), shows that the midbrain and left para-hippocampus presented consistently higher brain activity, across both comparisons (*p* < 0.001, uncorrected).

Additionally, consistently stronger activity was observed in the midbrain and left para-hippocampus during RC regardless of whether RC was compared with NRC or NoC (see Figure 5).

### Brain Asymmetry Results

**FIGURE 6:**
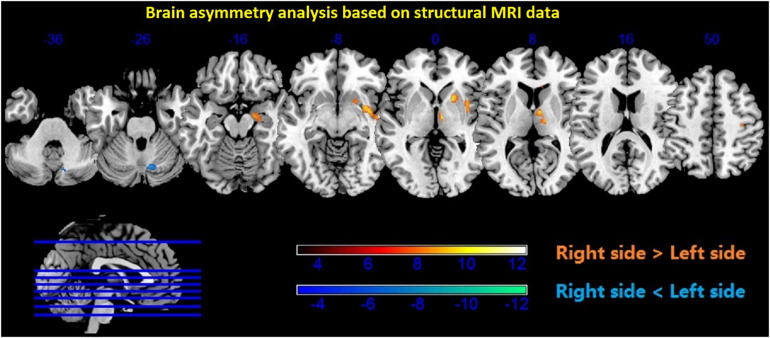
Brain asymmetry analysis showed higher density in the subcortical regions of the right thalamus, putamen, caudate, hippocampus, and amygdala (*p* < 0.001, uncorrected). Red color—right > left; blue color—left > right.

The participants with long-term RC experience showed a mainly rightward brain asymmetry, including the temporal lobe and the limbic system. Small differences were observed in the cerebellum as well (see Figure 6 and Table 2).

**TABLE 2:** Voxel-based analysis of the hemispheric asymmetries observed in the participants (*p* < 0.001, uncorrected).

Anatomical label (aal)	Peak *T*-value	Voxels	Co-ordinates		
Right > left					
Temporal_sup	12.29	1,273	57	−18	−6
Cerebelum_tonsil_R (Cerebelum_9_R)	7.79	245	11	−59	−59
Precentral gyrus	8.42	83	35	−20	54
Thalamus	9.36	251	8	−9	2
Putamen	10.5	193	20	11	−3
Hippocampus/Amygdala	7.48	71	18	−9	−17
Caudate	7.14	69	15	18	12
Left > right					
Sup_semilunar_R (Cerebelum_6_R)	−7.9	142	11	−69	−26
Cerebelum_biventral_R (Cerebelum_8_R)	−8.44	146	29	−53	−50

*L, left; R, right; Inf, inferior; Mid, middle; Post, posterior; Sup, superior.*

## Discussion

Emotionality is critically important for individuals with respect to their attainment of sentience, and emotion regulation is essential for interaction between members in a community (Rappaport and Corbally, 2016). The present study shows that religious activity affects brain regions involved in affective processing. It demonstrates that compared with either NRC or NoC, RC results in stronger engagement of subcortical regions in experienced participants and in asymmetric activation of the amygdalae. These patterns are particularly noticeable when participants are confronted with fearful events of negative valence. This finding is in line with a previous ERP study, which showed significant differences between RC and NRC during emotion regulation (Gao et al., 2017). The major involvement of subcortical regions, including the amygdala, para-hippocampus, and brainstem (pons) illustrate that RC can influence the function of these emotion-related brain regions in a direct manner. The effect of RC on these brain regions suggests that RC can induce a potent and pervading positive emotion. In the context of religious practice, this fits with the commonplace scenario, where a religious devotee uses RC when confronted by negative events, to invoke a strong positive emotion and counterbalance any negative effects.

Emotional processes are largely related to the activities of the limbic system, while the brainstem and cerebellum are also involved. Neocortical regions that evolved later in the frontal lobe can regulate and orchestrate, sometimes even voluntarily, the manifestation of emotional responses through interactions with subcortical regions (Damasio et al., 2000; Milardi et al., 2016). Given that human cognition tends to be negatively biased (Vaish et al., 2008), emotion regulation is important for psychological wellbeing as well as for social and emotional development. In addition, hormonal processes, especially related to the gonadal hormones, play a role in emotion regulation, through cyclic chemical messaging and its effect on mood (Graham et al., 2018).

This study demonstrated an asymmetric pattern of brain function and structure with regard to the effect of RC on fearful emotion regulation. Models of brain asymmetry in emotional processing have previously suggested that the processing of emotional events tends to be asymmetric, with left and right homotopic brain regions being differentially involved in processing emotional information (Alves et al., 2008). While engaged in RC, the right and left amygdala were both significantly more active during the viewing of fearful IAPS pictures (compared to viewing neutral IAPS pictures) but the left amygdala was activated more strongly and to a larger extent, with a significant cluster volume approximately 10 times larger than that of the right amygdala. The amygdalae were also activated, but to a lesser extent, when the participants engaged in RC without viewing any IAPS pictures, and in that condition the same asymmetric activation was observed. However, amygdala activity was not observed during any other condition. This indicates that amygdala activity is more pronounced while repetitively and religiously chanting “Amitābha,” especially under the most stressful condition of our experimental paradigm. The observed asymmetric activation suggests that the left amygdala may play a more critical role than the right amygdala in regulating stressful emotions during RC.

The functional asymmetry of the amygdalae found in the current study is consistent with previous evidence related to affective stimulation. A previous study reported that the left amygdala specialized in sustained emotional evaluation for both negative and positive events, whereas the right amygdala was more responsive to negative emotional stimuli (Wright et al., 2001). However, another fMRI study found that some emotion regulation strategies, including detachment, expression suppression, and distraction, are associated with lower activity in the left amygdala, while another emotion regulation strategy, that of reinterpretation, is not (Dorfel et al., 2014). Such a difference may imply a more complex role for the amygdala in emotion processing, because evaluative processing can influence amygdala activity and enable affective flexibility (Cunningham et al., 2008; Cunningham and Brosch, 2012). It is worth noting that repetitive mental training can also enhance the neural efficiency of the neural network that is relevant to the task being trained (Whatmough et al., 2008; De Brigard et al., 2017).

Our study demonstrated that repetitively and religiously chanting “Amitābha” results in increased brain activity, especially in the left amygdala. The activity of the left amygdala increased further when participants were confronted with stressful stimuli. Correlation analysis revealed a significant association of amygdala activation levels solely between RC and RC-Fear, but not between RC and RC-Neut. This indicates that RC may directly affect the reactivity of the amygdalae, especially the left amygdala, and this may form the mechanistic explanation for the effect of RC on emotion regulation. A previous study suggested that the left amygdala is responsive to the arousal dimension of emotion; that is, that stimulation of the left amygdala can result in either positive (e.g., joy) or negative (e.g., fear) emotional states, whereas the right amygdala is only responsive to stimuli with negative valence, such as sad or disgusting pictures (Wright et al., 2001).

In addition, other subcortical regions, including the thalamus, para-hippocampus, and brainstem, showed high activity in the RC-Fear condition. Interestingly, increased activity during RC was also evident in the midbrain, in the dorsal part of the pons. This part of the brainstem consists of structures involved in affective processes, including the locus coeruleus and parabrachial complex. These structures are involved in suppressing fear and modulating pain in humans (Mobbs et al., 2007). The locus coeruleus has projections to a broad network of brain regions, including the amygdala and thalamus, and it is involved in enhancing emotion-related memory (Tully and Bolshakov, 2010). The parabrachial complex is involved in gathering sensory inputs and conveying them to emotion-related regions like the amygdala, the thalamus, and the hypothalamus. Through such mechanisms, the parabrachial complex is involved in modulating the arousal level of elicited emotions (Edlow et al., 2012).

The thalamus plays an intermediary role in relaying emotional information from the brainstem to rostral brain structures. For example, the ventral posteromedial nuclei of the thalamus receive information from the parabrachial complex and convey it to higher structures such as the insular cortex and forebrain regions (Venkatraman et al., 2017). During RC, the thalamus may be involved in transmitting emotional signals from the brainstem to the forebrain regions and may modulate the subjective “feeling” sensation of an emotion (Venkatraman et al., 2017).

In our study, further findings resulted from the analysis of the brain structure, showing anatomical asymmetry in the thalamus, putamen, caudate, hippocampus, amygdala, and cerebellum. According to a putative biological model of depression, these brain regions form a network related to biased memory (Disner et al., 2011). In the case of repetitive RC, RC is biasing memory formation in a positive way through an emotional schema with religious connotations, as the devotees usually generate a feeling of bliss and serene joyfulness when chanting the name “Amitābha” repeatedly, as taught in the Amitābha Buddha Sutra (Hsuüan and Buddhist Text Translation Society, 2003; Halkias and Payne, 2019).

With long-term repetitive RC, it is assumed that the repetitive generation of such an emotional schema with religious connotations facilitates its activation and maintenance (Lau, 1989; Xu, 2019). Emotional maintenance is one of the three main modes of emotion regulation, with the other two being upregulation and downregulation (Gross, 1998). The fMRI results of this study showed higher activity in the medial prefrontal cortex (mPFC) during RC, which can help maintain an emotional state (Waugh et al., 2014). Maintaining an emotional state can improve the performance of an individual on emotion-related tasks (Tamir et al., 2008). It helps individuals to orient their attention to the external environment, during fearful situations, in order to monitor, plan, and execute ideas (Hanakawa et al., 2008). This is in line with the Pure Land doctrine, dictating that practitioners should have a compassionate intention to help themselves and others in a frightening situation. The mPFC has multiple functions, including the control of cognition, pain, and affect. It is also related to reward, social processing, and episodic memory (de la Vega et al., 2016). There are interactions between the PFC and other brain regions, notably including subcortical regions. For example, strong connections between the medial frontal lobe and the amygdala were found in macaque monkey and human studies (Amaral and Price, 1984). Furthermore, the PFC may play a mediative role in pain detachment and emotion modulation during religious practice (Wiech et al., 2008; Harris et al., 2009).

RC-Fear also induced greater activity in the bilateral occipital region, parietal lobe, and other subcortical regions of the brain, compared with NRC and NoC. It could be that RC becomes related to a strong and positive spiritual feeling through long-term practice. RC could thus habitually activate a pattern of activity in a specific brain network, as the imagery of a believed supernatural agent may activate a pathway between the right lateral temporal areas and occipital areas (Kapogiannis et al., 2014). The occipital activity suggests that the participants were not shifting their attention away from the negative stimuli, but rather paying more attention to them during RC. This rules out attention distraction as a strategy of emotion regulation during RC.

The MRI analysis of anatomical asymmetry also illustrates inter-hemispheric differences in the superior temporal lobe, precentral region, and cerebellum, and these regions are involved in vocal movement and auditory information processing. In daily living, the devotees chant loudly for a long time and the long-term practice may enable the practitioner to quickly generate an image of the Amitābha Buddha while inducing positive emotion and hyperactivity of the amygdala. This can enhance the bottom-up regulation of the hippocampus to imagine the spatial layout of the Pure Land and form a religious schema with enhanced positive emotion. The putamen and caudate can be involved in this process, given their critical role in skill learning. Long-term practice of RC may allow the Pure Land Buddhism devotees to recall without the need to recruit top-down regulation from regions associated with higher cognition like the PFC (Disner et al., 2011).

Several limitations of the present study are worth noting. First, this study had no control group comprised of non-religious participants or participants with different degrees of experience of RC. Therefore, the presented data cannot be informative with regards to whether RC exerts similar effects in people with no previous practice. Furthermore, it is not clear to what extent being a religious devotee is a prerequisite for such effects, or to what extent any person engaging in RC would present similar brain activations and in the long-term similar structural brain asymmetry. Second, it is possible that gonadal hormones and menstrual cycles may have affected brain activation patterns in female participants during the experiment (see Supplementary Table 1). Thus, this should be controlled in the future. Third, the reported results comprised statistical tendencies rather than strongly significant findings (e.g., Supplementary Tables 4, 5). Thereby, the present results should be considered with caution as preliminary and the effect of RC on negative emotion regulation must be investigated further. Future studies with *in vivo* measurement of neurotransmitters and modulators, that are feasible with advanced proton magnetic resonance spectroscopy (H-MRS) techniques (Wijtenburg et al., 2015), may clarify more directly the neural underpinnings of RC, especially if performed according to a Randomized Controlled Trial design.

In summary, our multi-modal functional and structural MRI data suggest that repetitive RC can influence the brain both functionally and structurally. Functionally, RC activates a wide network of brain regions, including the amygdala and midbrain, as well as the frontal and occipital areas. This brain network may enable the formation of religious schemas associated strongly with positive emotion. With long-term practice, the brain may undergo asymmetric structural changes in the subcortical regions of thalamus, putamen, caudate, amygdala, and hippocampus, as well as in the temporal and precentral gyri, as evidenced by the present study. Overall, the present study elucidates neural mechanisms in support of the position that RC can provide a powerful and unique method for emotion regulation. RC appears to modulate emotion by directly engaging key subcortical areas, including the amygdala, as well as neocortical areas, including the mPFC, to sustain and prolong a positive emotional state; thereby counterbalancing the impact of negative events. The tentative interpretations offered here open up experimental avenues for investigating the therapeutic efficacy of RC in alleviating the symptoms of emotional distress.

## Data Availability Statement

The datasets presented in this article are not readily available because of their file size. Requests to access the datasets should be directed to HS, hinhung@hku.hk.

## Ethics Statement

The studies involving human participants were reviewed and approved by the Human Research Ethics Committee of the University of Hong Kong. The patients/participants provided their written informed consent to participate in this study.

## Author Contributions

JG designed the experiment, analyzed and interpreted the data, and wrote the initial manuscript. SS offered advice on the experimental design, helped interpret the data, and revised the manuscript. HL assisted in data analysis, interpretation of data, and manuscript revisions. BW assisted in manuscript revisions. HW worked on the brain asymmetry analysis. CC helped designing the experiment, gave suggestions on data analysis and manuscript revisions. HS helped designing the experiment and manuscript revisions. All authors contributed to the article and approved the submitted version.

## Conflict of Interest

The authors declare that the research was conducted in the absence of any commercial or financial relationships that could be construed as a potential conflict of interest.

## Footnotes

1 http://marsbar.sourceforge.net

2 www.ibm.com/products/spss-statistics

3 www.fil.ion.ucl.ac.uk/spm

4 http://dbm.neuro.uni-jena.de/vbm/download/

5 www.mccauslandcenter.sc.edu/mricro/mricron
