# Supplementary figures and images for: Repetitive Religious Chanting Invokes Positive Emotional Schema to Counterbalance Fear: A Multi-Modal Functional and Structural MRI Study
Source: Front Behav Neurosci. 2020 Nov 24;14:548856. doi: 10.3389/fnbeh.2020.548856 (PMC7732428; doi:10.3389/fnbeh.2020.548856)

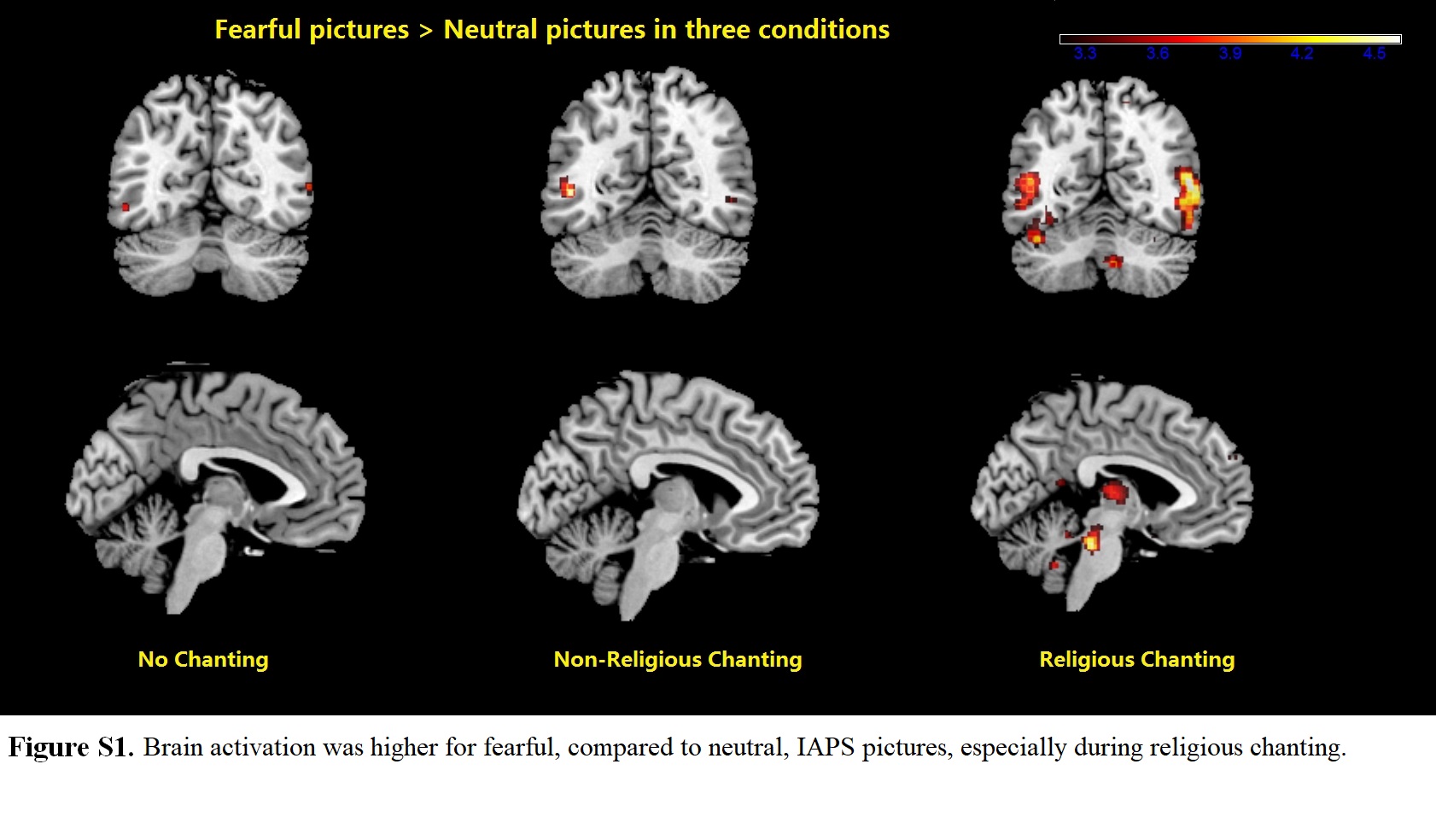

Supplement: Supplementary file 1 [file Image_1.JPEG]

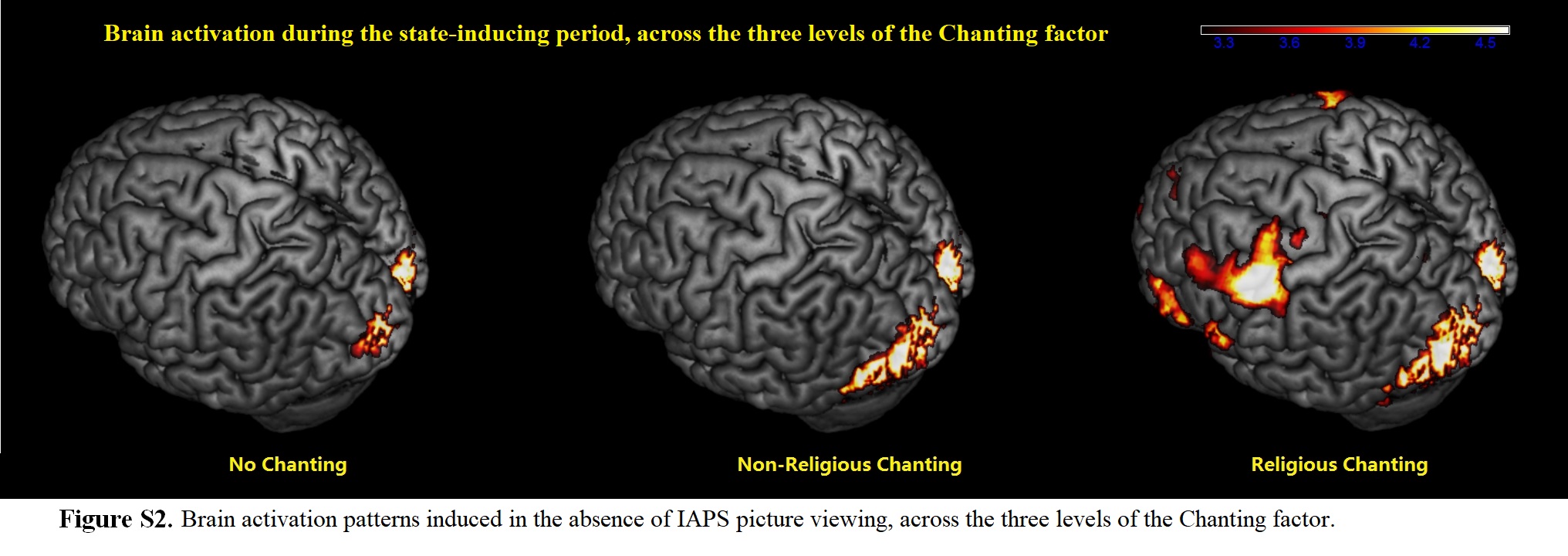

Supplement: Supplementary file 2 [file Image_2.JPEG]
