# Supplementary material for: Repetitive Religious Chanting Invokes Positive Emotional Schema to Counterbalance Fear: A Multi-Modal Functional and Structural MRI Study
Source: Front Behav Neurosci. 2020 Nov 24;14:548856. doi: 10.3389/fnbeh.2020.548856 (PMC7732428; doi:10.3389/fnbeh.2020.548856)
Supplement: Supplementary file 3 [file Table_1.pdf]

Appendix

sTable 1. Behavioral analysis based on participants' ratings, in response to the following 10 questions and in relation to the figure displayed during the state-inducing period (i.e. Amitābha Buddha, Santa Claus, or colour-balanced picture) in each of the three levels of the Chanting factor (RC, NRC and NoC). Responses were measured on a likert scale with 1 representing the minimum and 9 representing the maximum.

Q1. How strong was your **faith** in the figure of the preceding trial?

Q2. How **happy** did you feel when you looked at the figure in the preceding trial?

Q3. How **peaceful** did you feel when you looked at the figure in the preceding trial?

Q4. How **merciful** did you feel while looking at the figure in the preceding trial?

Q5. How **excited** were you while looking at the figure in the preceding trial?

Q6. Was your **mind wandering** in the preceding trial?

Q7. Did you have any **visualization** while looking at the figure in the preceding trial?

Q8. Did you feel **sleepy** during the preceding trial?

Q9. Have you been able to **concentrate** during the preceding trial?

Q10. How **fast** was your chanting during the preceding trial?

sTable 2. Statistical result of participants' ratings of the chanting figure used in each chanting condition (i.e. images of 1, Amitābha Buddha; 2, Santa Claus; and 3, colour-balanced control picture). Post-hoc comparisons between conditions were Bonferroni corrected.

Question	Condition	Mean	Std. Deviation	Post-hoc	Comparison	Mean Difference	p-value (Bonferroni corrected)
Q1	1	8.16	0.958	1	2	4.895*	**<0.001**
**faith**	2	3.26	2.557	1	3	6.211*	**<0.001**
	3	1.95	2.094	2	3	1.316	0.138
Q2	1	7.32	1.797	1	2	1.316	0.090
**happy**	2	6.00	1.528	1	3	4.368*	**<0.001**
	3	2.95	2.094	2	3	3.053*	**<0.001**
Q3	1	7.72	1.447	1	2	1.827*	**0.016**
**peaceful**	2	5.89	1.883	1	3	1.617*	**0.040**
	3	6.11	2.307	2	3	-0.211	1.000
Q4	1	7.94	1.305	1	2	4.260*	**<0.001**
**merciful**	2	3.68	2.770	1	3	5.313*	**<0.001**
	3	2.63	2.385	2	3	1.053	0.468
Q5	1	5.237	2.4402	1	2	2.6053*	**0.001**
**excited**	2	2.632	2.1398	1	3	3.7632*	**<0.001**
	3	1.474	1.3068	2	3	1.1579	0.249
Q6	1	4.763	2.6054	1	2	0.7368	1.000
**mind wandering**	2	4.026	2.3480	1	3	1.0965	0.541
	3	3.667	2.4010	2	3	0.3596	1.000
Q7	1	5.324	2.7326	1	2	1.7647	0.177
**visualization**	2	3.559	2.8716	1	3	3.1471*	**0.004**
	3	2.176	2.3515	2	3	1.3824	0.409
Q8	1	6.44	2.479	1	2	3.023*	**0.004**
**sleepy**	2	3.42	3.203	1	3	4.167*	**<0.001**
	3	2.28	2.372	2	3	1.143	0.621
Q9	1	4.605	3.3606	1	2	0.8158	1.000
**concentrate**	2	3.789	2.6994	1	3	1.6579	0.246
	3	2.947	2.5270	2	3	0.8421	1.000
Q10	1	7.16	1.979	1	2	0.263	1.000
**fast**	2	6.89	1.792	1	3	2.269*	**0.010**
	3	4.89	2.847	2	3	2.006*	**0.026**

sTable 3. Compared to female participants, male participants had a tendency for higher brain activity in the right frontal cortex during negative picture viewing (p < 0.05, uncorrected). This comparison was requested by a reviewer.

Anatomical label (aal)	Peak T-value	Voxels	Co-ordinates
Male > female			
Frontal_Inf_Oper_R	5.40	18	54 6 24
Frontal_Inf_orb_R	4.08	12	48 30 -4

sTable 4. Comparison of fearful picture viewing (in contrast to neutral picture) between religious chanting and Non-religious chanting conditions, and between religious chanting and No-chanting conditions. (k > 20, p < 0.05, uncorrected).

**Anatomical label (aal)**	**Peak T-value**	**Voxels**	**Co-ordinates**
**RC(Fear-Neut) > NoC(Fear-Neut)**			
Cerebelum_3_L	3.85	37	-6 -34 -22
Fusiform_L	3.54	21	-26 -32 -26
**RC(Fear-Neut) > NRC(Fear-Neut)**			
Vermis_1_2	3.73	32	2 -30 -24
ParaHippocampal_L	3.5	22	-24 -26 -16
Precuneus_L	3.47	21	-10 -48 12

sTable 5. Brain activity during each chanting condition in the abence of visual stimulation with IAPS pictures, compared to baseline (k > 20; p < 0.001, uncorrected).

Anatomical label (aal)	Peak T-value	Voxels	Co-ordinates
**religious chanting**			
Fusiform_R	12.14	13205	40 -48 -18
Postcentral_L	7.14	1634	-52 -6 44
Frontal_Inf_Tri_R	5.48	378	52 42 4
Supp_Motor_Area_L	5.09	100	-2 0 58
Precentral_R	4.94	980	58 -6 42
Temporal_Pole_Sup_L	4.85	138	-56 16 -4
Frontal_Inf_Orb_L	4.49	352	-50 40 -4
Vermis_9	4.26	46	2 -62 -40
ParaHippocampal_L	4.07	42	-10 -26 -12
Occipital_Sup_R	3.96	65	22 -62 40
Precentral_L	3.9	53	-30 -20 64
Frontal_Inf_Oper_R	3.83	23	58 22 32
Hippocampus_L	3.78	23	-22 -26 -6
Frontal_Sup_Medial_L	3.7	33	0 56 34
**Non-religious chanting**			
Occipital_Inf_R	9.85	10398	32 -80 -12
Frontal_Mid_Orb_R	4.65	109	42 56 -2
Precentral_R	4.23	74	64 2 24
Precentral_R	3.91	28	56 -8 42
Hippocampus_R	3.85	20	26 -28 -4
**No-chanting**			
Lingual_L	8.89	4682	-6 -84 -8
